# A Cognitive Occupation-Based Programme for People with Multiple Sclerosis: A Study to Test Feasibility and Clinical Outcomes

**DOI:** 10.1155/2018/1614901

**Published:** 2018-05-02

**Authors:** Sean Reilly, Sinéad M. Hynes

**Affiliations:** Discipline of Occupational Therapy, National University of Ireland Galway, Galway, Ireland

## Abstract

Cognitive impairments are common in MS and affect personal, social, and occupational functioning. There is a developing body of evidence highlighting the role of cognitive rehabilitation, but there is still no evidence for a validated holistic approach. The aim of this study was to assess the effectiveness of Cognitive Occupation-Based Programme for People with Multiple Sclerosis (COB-MS) for improving daily life and cognitive impairment. This study used an experimental pretest/posttest design with eight-week follow-up. Participants were recruited from MS networks using convenience sampling. The primary outcome measure was the GAS. Secondary outcomes included the OSA-DLS, CVLT-II, BVMT-R, SDMT, TMT, BRIEF-A, and EMQ-R. Twelve participants were recruited, aged 39–73 years (mean: 55.08; SD: 9.61). There were statistically significant improvements in the GAS (*p* < .002), CVLT-II: total free recall (*p* < .000), short delay free recall (*p* < .018), long delay free recall (*p* < .008), BVMT-R total recall (*p* < .000), TMT part B (*p* < .044), and EMQ-R (*p* < .006). Except for the BRIEF-A, clinically significant improvements were observed in secondary outcome measures at posttest and follow-up. Limitations include selection bias and subtle practice effects in cognitive measures. Results suggest that a larger scale study is justified considering improvements seen in daily life and cognitive measures.

## 1. Background

Multiple Sclerosis (MS) is a demyelinating disease of the central nervous system, characterised by the development of lesions in the brain and spinal cord [1]. According to Amato et al. [2], the prevalence of cognitive difficulties in MS ranges from 43% to 70%. The most commonly affected cognitive domains in people with MS are memory, attention, processing speed, and executive functions [3]. Cognitive difficulties affect the daily lives of people with MS in several ways. Cognitive impairment resulting from MS has been found to be associated with impaired social functioning [4], reduced employability [5], and decreased quality of life [6].

Cognitive rehabilitation (CR) is an intervention that aims to improve cognitive impairment through using compensatory and restorative approaches [7]. There are many unique factors to be considered when developing interventions for people with MS such as the disease's fluctuating nature, associated fatigue, and the emotional well-being of the person [8]. To date, most studies examining CR in MS have mainly involved computer-based interventions [9–12], although others have investigated non-computer-based (face to face) interventions that use educational methods [13, 14]. Research has found that computer-based interventions, specifically targeting the domain of attention (e.g., [9, 10]), are effective at improving outcomes on cognitive measures for people with MS. Computer-based interventions that target several cognitive domains [15] also found significant improvements in objective and subjective cognitive outcome measures. In contrast, many computerised interventions that specifically target memory did not lead to improvements (e.g., [16, 17]). It is apparent that non-computer-based interventions are more beneficial for improving memory [18] and several cognitive domains [19, 20] than computerised interventions. Group interventions and frequent professional contact appear to be important components in the effectiveness of CR for people with MS.

Despite the findings from individual studies being generally positive, the pooled results of a meta-analysis in a Cochrane review [21] suggest that the effectiveness of cognitive rehabilitation for people with MS is far from being conclusive. In two recent reviews [22, 23], the authors found low-level evidence that CR reduces cognitive difficulties in MS. Both reviews concluded that CR in MS is in its relative infancy. There is a clear need for more randomised control trials of sound methodological quality before conclusions can be drawn on the possibility of improving function through CR. In addition, both reviews recommended that future studies should examine interventions that aim to increase occupational participation and use outcomes that measure impact on daily life—cognitive interventions adopting a specific approach may not necessarily generalise to functional domains. Findings and recommendations provided the rationale for the current study.

From the current evidence and recommendations provided in the literature, there was a clear need for a holistic cognitive intervention targeting several domains using a functional approach that is measured by and focuses on occupational participation. A Cognitive Occupation-Based Programme for People with Multiple Sclerosis (COB-MS) was developed in response to this noted clinical treatment gap, and it focuses on facilitating people with MS to engage more effectively in everyday occupations that they find difficult as a result of their cognition [24].

Thus, the purpose of this current study was to establish clinical outcomes of COB-MS in relation to daily life and cognition. This study will contribute towards COB-MS' proof of principle and add to the paucity of research in the field of MS and CR.

Specifically, the following research questions were addressed:

- Will people who receive COB-MS have improved daily life scores?
- Will people who receive COB-MS see improvements in their cognition?

We were interested if any gains seen were also maintained at follow-up of 8 weeks.

## 2. Method

### 2.1. Recruitment

Information regarding the study was available to participants through MS networks such as the monthly MS Ireland newsletter, biannual MS Ireland eZine, and research section of MS Ireland website and Facebook page. Recruitment posters were also hung, with permission, in the outpatient department of University Hospital Galway. Study details and the second author's contact information were available on all recruitment material.

### 2.2. Participants

Participants were self-selected by contacting the second author via phone or email if they were interested in taking part. To be part of the convenience sample, participants had to meet the following inclusion criteria: (i) be aged 18 years of age or older, (ii) be fluent in written and spoken English, (iii) have a diagnosis of MS, (iv) have mild cognitive difficulties as shown by a score of >22 on the MSNQ, (v) be clinically stable, (vi) can provide informed consent, (vii) do not have neurologic history other than MS, including evidence of current dementia, (viii) do not have history of major depressive disorder, schizophrenia, or bipolar disorder I or II, (ix) do not have history of diagnosed substance use or dependence disorder, and (x) live in the community. Exclusion criteria were the following: (i) cognitive impairment that would affect reliable participation or capacity to give informed consent and (ii) being incarcerated or institutionalised.

Eligibility was determined by the second author during the initial contact. If deemed ineligible, the participant was thanked for their time and the reasons were explained. For eligible participants who were satisfied with the study requirements, verbal consent was sought and their availability was noted to allow for scheduling of COB-MS sessions. Once individual and group sessions were confirmed by the second author, the first author then contacted the participants via phone to schedule the baseline assessment. The first author posted an information sheet to participants for the study. The first author also went through the information sheet with participants at the beginning of the baseline assessment and answered any questions the participants had before signing the written consent form.

### 2.3. COB-MS Programme

The COB-MS programme consists of eight sessions: two being individual and six being group-based. The focus of the COB-MS is on managing the demands of employment and daily life by using compensatory strategies and routines and learning new techniques that can be integrated into daily occupations and contexts in order to make it meaningful to the participant. The programme was facilitated by the second author. The programme consisted of eight sessions over 9 weeks, beginning one week after baseline assessments and finishing one week before posttest assessments. The COB-MS programme takes a three-pronged approach to cognitive rehabilitation using education, remediation, and adaption to help people meet their goals while managing their cognitive challenges. The programme is informed by the Person-Environment-Occupational Performance model [25].

Table 1 summarises the content of each of the 60-minute sessions.

### 2.4. Outcome Measures

Participants completed a total of eight assessments. The Goal Attainment Scaling (GAS) [26] was the primary outcome measure of this current study and allowed participants to set meaningful goals related to daily life, which could be measured in a systematic way. Given the emphasis placed on goal-setting in COB-MS and the importance of personal goals as a source of motivation for participants, goal achievement reflected the practical benefit of COB-MS.

Secondary outcomes included measures of occupational competence [Occupational Self-Assessment-Daily Living Scales (OSA-DLS) [27]], verbal memory [California Verbal Learning Test-II (CVLT-II) [28]], visual memory [Brief Visuospatial Memory Test-Revised (BVMT-R) [29]], processing speed [Symbol Digit Modality Test (SDMT) [30]], divided attention [Trail Making Test (TMT) [31]], perceived executive functioning [Behaviour Rating Inventory of Executive Function-Adult Version (BRIEF-A) [32]], and self-reported memory difficulties [Everyday Memory Questionnaire-Revised (EMQ-R) [33]].

Data was collected by the researcher at three time points: one week before the first COB-MS session (baseline), one week after the final COB-MS session (posttest), and eight weeks after the final COB-MS session (follow-up). Except for the GAS, all assessments were administered at three time points, with alternate forms used where possible.

### 2.5. Statistical Analysis

SPSS Version 23 was utilised to analyse the data. Descriptive statistics were used to describe the characteristics of the sample regarding gender, age, education, work status, MS type, disease duration, and MSNQ score. To answer the research questions, inferential statistics were utilised. The Friedman test measured changes in mean daily life and cognitive scores over three time points, whereas the Wilcoxon signed-rank test measured changes over two time points.

## 3. Results

In total, 1 male and 11 female participants received COB-MS. The types of MS varied between participants and included relapsing remitting (*n* = 5) or primary progressive (*n* = 3) types. Four participants were unsure of their MS type. Participants' work status also varied and included long term disability (*n* = 5), staying at home parent (*n* = 3), or being retired (*n* = 4).

A full data set was obtained, as the twelve participants completed outcome measures at each of the three time points. Descriptive statistics were expressed as a mean, standard deviation (SD), minimum, and maximum.  Table 2 illustrates the demographic data of the sample in relation to age, disease duration, education, and MSNQ score.

### 3.1. Daily Life

After receiving COB-MS, participants' goal achievement improvement in scores on the GAS (Table 3) was statistically significant (*z* = −3.061; *p* = .02). This shows that the meaningful goals set in relation to daily life were achieved by follow-up.  Table 4 shows that occupational competence scores on the OSA-DLS were clinically significant as posttest and follow-up scores were greater than baseline.

### 3.2. Cognition

Findings from cognitive outcome measures are shown in Table 5. Statistically significant improvements were observed in verbal memory on the CVLT-II: in total free recall (*p* = .000), short delay free recall (*p* = .018), and long delay free recall (*p* = .008). In visual memory, a statistically significant difference was obtained on the BVMT-R in total recall (*p* = .000). In part B of the TMT, improvements in divided attention were statistically significant (*p* = .044). Although results for part A of the TMT and the SDMT were not statistically significant, results show that processing speed improved across the three time points. Participants reported significantly fewer memory difficulties in daily life on the EMQ-R (*p* = .006). In contrast to the trend of results, perceived executive functioning scores on the BRIEF-A at follow-up did not improve when compared to baseline.

### 3.3. Post Hoc Tests

After establishing that there were statistically significant differences somewhere among the three time points in Friedman test results for the CVLT-II, BVMT-R, TMT, and EMQ-R, post hoc tests were used to identify changes between individual time points (Table 6). Wilcoxon signed-rank tests were used to compare Time 1 with Time 2, Time 2 with Time 3, and Time 1 with Time 3.

To control for Type 1 errors, a Bonferroni adjustment was applied to the alpha values. This involved dividing the original alpha level of .05 by the total number of tests used (.05/3 = .01666). Thus, the adjusted alpha level for determining statistical significance was .0167.

## 4. Discussion

Results suggest that people who receive COB-MS have significantly higher mean daily life scores at posttest and eight-week follow-up. The GAS, which was the primary outcome measure of the study, showed a statistically significant increase in goal achievement with a large effect size. This adds to the findings of Mantynen et al. who observed similar changes in the GAS and showed that personal goals for their computer-based and non-computer-based interventions were well achieved [34]. However, it is important to note that the goals set by participants in this study related to leisure, self-care, and productivity occupations in their daily lives and not just the intervention as seen in Mantynen et al.'s work [34]. Therefore, the improvements observed in the GAS in this study reflect the practical benefit of COB-MS and acknowledge the real-world implications of COB-MS in daily life.

Although there was no statistically significant increase in the OSA-DLS, scores at posttest and follow-up were greater than baseline and are indicative of clinical significance. With regard to the trend of competence scores in the OSA-DLS, it appears that people who receive COB-MS feel that they improve their ability to sustain a pattern of occupational behaviour, which is both satisfying and productive. Therefore, the perceived ability to perform activities of daily living (ADLs) and instrumental activities of daily living (IADLs) increased after receiving COB-MS. Similarly, the importance placed on daily life occupations was also greater at posttest and follow-up. Unfortunately, no comparisons with other studies can be made as the OSA-DLS was not used as an outcome measure in previously reviewed literature. However, there is an abundance of literature pertaining to the Model of Human Occupation which illustrates the meaning of the OSA-DLS results [35]. It has been stated by Lee et al. that a person's motivation for occupation, often referred to as volition, is influenced by personal causation, values, and interests [36]. Volition is also recognised as a fundamental factor in the achievement of goals. Thus, this is in line with findings in the current study as higher competence scores (personal causation) and importance scores (values and interests) appear to have contributed towards people achieving their daily life goals after receiving COB-MS.

With regard to cognition, results suggest that people who receive COB-MS have statistically significant improvements in verbal memory scores at posttest and follow-up, as shown on the CVLT-II. This finding is similar to verbal memory improvements seen in computer-based interventions [11, 37], non-computer-based interventions [38, 39], and studies that investigated computer-based and non-computer-based interventions [40, 41]. While one may think that an intervention that specifically trained memory would result in better verbal memory outcomes than a nonspecific intervention, this is not the case. For example, two computer-based interventions specifically targeting memory failed to find statistically significant improvements in verbal memory [12, 17], whereas the nonspecific intervention in this current study did. This observation supports the argument that interventions for people with MS are more effective in the presence of a healthcare professional [20].

Findings from this current study also suggest that people who receive COB-MS improve statistically in visual memory, as shown in the BVMT-R. These findings are reminiscent of those by computer-based interventions [15, 40] and interventions using a combination of computer-based and non-computer-based training [37, 41, 42]. Once again, there is evidence supporting the argument by Jonsson et al., as interventions that involved frequent input from a healthcare professional appear to be more effective for improving visual memory [39]. It appears that regular contact with a professional is required as people may not comply with the intervention if they are not monitored weekly [14]. In addition, qualitative research suggests that people enjoy the social element of group interventions [13], which is absent in individual and computer-based interventions. In COB-MS, each session involves discussion around cognitive difficulties, whereby the facilitator explains various strategies and techniques that can be applied to improve occupational performance in daily life. A group-based design is a feasible and cost-efficient method to deliver interventions in a healthcare setting, making the COB-MS meaningful to frontline health professionals. The format of the COB-MS, importantly, allows for individualisation through the two individual sessions coupled with goal setting.

Although results pertaining to processing speed and working memory on the SDMT did not improve statistically, they were nonetheless clinically significant. From the literature reviewed which utilised the SDMT as an outcome measure, no study identified a statistically significant improvement [9, 10, 14, 16, 38]. However, it is interesting to note that people who received COB-MS improved in the SDMT at each time point (follow-up > posttest > baseline). Considering that there are minimal practice effects in the SDMT [43], it would appear that the 8-week gap between posttest and follow-up allowed people to integrate and generalise strategies learned in COB-MS into their daily lives. This finding validates the purpose of a follow-up assessment in the current study.

There was also a clinically significant improvement for processing speed in part A of the TMT. Similar to the SDMT, participants performed better at each time point. It must be noted that, at one-month intervals, part A is susceptible to practice effects after the first trial [44]. However, according to the same research by Craddick and Stern [44], there were no practice effects for part B in the current study as it was only utilised at three time points. Thus, it was positive to discover statistically significant improvements for divided attention in part B after receiving COB-MS. While clinical improvements in the TMT were frequently observed in the literature [10, 34, 41], few studies identified statistically significant changes. Although Amato et al. identified statistically significant improvements in both parts of the TMT, their computer-based intervention specifically targeted attention, which means that the results were unsurprising [9].

Results suggest that people who receive COB-MS perceive fewer difficulties with memory in daily life. Although a statistically significant improvement was observed after the Friedman test, the improvement was not statistically significant when using a Bonferroni adjustment. This is similar to Carr et al. who also identified a clinically but not statistically significant improvement [13]. It is possible that the small sample size in both studies may have led to the lack of a statistically significant difference. Furthermore, the use of a Bonferroni adjustment to reduce the chance of Type 1 error made the significance level more conservative. However, it is correct practice to use a Bonferroni adjustment when making multiple comparisons.

Although participants perceived fewer difficulties with executive functioning at posttest, scores at follow-up show that there was a greater frequency of difficulties reported in comparison with baseline. However, similar to the OSA-DLS, it is difficult to draw comparisons as the BRIEF-A was not used as an outcome measure in previously reviewed literature. While some studies have identified statistically significant improvements in objective executive functioning [41, 42], it has been argued by Sullivan et al. that subjective cognitive functioning often correlates poorly with the objective equivalent [45]. It may have been that, given the period of reflection that comes with follow-up assessment, participants became more aware of the metacognitive domains (e.g., planning and monitoring) and their difficulties in these areas. The subjective scores they provided may not necessarily reflect objective measurement but an increased insight and awareness of difficulties.

The results from this study suggest that people who receive COB-MS improve significantly (statistically or clinically), in all daily life outcome measures and most cognitive outcome measures, at posttest and follow-up. These findings are encouraging given the fact that this was a pilot study with a small number of participants. Considering that a nonspecific intervention was used in this study, it is not surprising that there were only two statistically significant improvements in cognitive outcome measures. As MS is a degenerative disease, cognitive difficulties are likely to worsen [1]. Therefore, it could be argued that a nonspecific intervention that addresses daily life, by targeting difficulties with cognition, will produce longer lasting benefits for people with MS rather than attempting to improve a specific cognitive domain. Thus, the validity of COB-MS is supported by statistically significant improvements in the primary outcome measure, the GAS, and clinically significant improvements in the OSA-DLS. However, there are some factors that limit the generalisability of findings.

Firstly, there is a high risk of self-selecting bias in this study. Therefore, the participants may have been highly motivated to learn and apply strategies from COB-MS in their daily lives. However, treatment contamination is incredibly difficult to control when recruiting community-dwelling participants. Although the prevalence of MS is reported to be three times more common in females [46], there was only one male participant and this limits generalisability to males. The lack of disease course data on the entire sample as well as the substantial variation in disease duration is also noted as a limitation. Another significant limitation that relates to participant selection is the use of the MSNQ. This measurement device was used as a self-report screening tool for cognitive impairment as part of the inclusion criteria, whereby participants required a score of at least 22 or more. However, the observation of ceiling effects in the results suggests that this cut-off score may have been too low, and previous studies have shown weak correlations between subjective and objective cognitive impairments [47, 48]. Nonetheless, it was decided to include people who had self-reported cognitive impairment (as shown in the MSNQ) without objective evidence because (i) this was a pilot study, which aimed to ensure that COB-MS was feasible in the largest possible sample of people with MS, (ii) some people may have experienced cognitive difficulties in daily life but would not be classified as impaired on an objective measure, and (iii) it was felt that the people would be more motivated to learn if they felt they had cognitive difficulties.

Secondly, despite using alternative assessment forms (CVLT-II and BVMT-R) and research stating minimal practice effects (SDMT and TMT part B), subtle practice effects cannot be completely ruled out in cognitive outcome measures. There are two types of practice effects: “test-specific,” which refers to people knowing the assessment format, and “item-specific,” which refers to people learning the actual content of the assessment (e.g., word lists or geometric figures [49, 50]). Although item-specific practice effects were mitigated by using alternative forms, test-specific practice effects were unavoidable. For example, at baseline assessments, participants were unaware that there was a delayed recall in the CVLT-II. However, at posttest and follow-up, it is likely that participants knew that they would have to recall the words and may have been internally listing them. In addition, the use of nonparametric tests to analyse outcomes is a limitation. The Friedman test and Wilcoxon signed-rank test are less sensitive than the equivalent parametric techniques, one-way repeated-measures ANOVA and paired-samples *t*-test, respectively. Thus, it is possible that statistically significant differences may have been missed [51].

## 5. Conclusion

Future research should use outcome measures related to daily life. It was highlighted in the literature review that it is unclear whether improvements in cognitive outcome measures are indicative of improvements in daily life [52]. The use of daily life assessments would also facilitate comparisons between studies, an issue that was observed in the current study. In addition, subjective cognitive assessments should continue to be used. Although Sullivan et al. argue that subjective and objective cognitive assessments correlate poorly with each other [45], findings from this current study suggest that they trend in the same direction. It appears that different concepts are being measured, with objective tools measuring how a person performs in a structured test, whereas subjective devices measure how a person feels they perform in daily life. Furthermore, outcome assessors should be blinded, and a follow-up period of approximately 6–12 months should be used to determine if improvements are maintained over the long term.

Occupational therapists and people with MS reported that COB-MS may be more beneficial for people who are newly diagnosed [24]. Thus, future studies of COB-MS could consider comparing its effectiveness for people who are newly diagnosed versus people who have been diagnosed for some time. Considering that Chiaravalloti et al. [18] found that people with moderate cognitive impairment benefitted more than those with mild cognitive impairment, it may be worth comparing the effectiveness of COB-MS for a group of moderately versus mildly impaired participants. These recommendations could also be integrated into occupational therapy practice, whereby COB-MS is provided to people with MS who are newly diagnosed and/or have similar levels of cognitive impairment. Previous studies found that older participants were more adherent in completing CR at home than younger participants [15, 41]. Therefore, future COB-MS studies should monitor completion of homework activities to determine adherence to the programme. In addition, the paucity of qualitative research indicates that participants of future COB-MS studies should be interviewed to determine benefits of the programme that cannot be detected in quantitative measures, as shown in Carr et al. [13].

The primary implication for occupational therapy practice is that this study provides an occupation-based intervention that is specific to the cognitive difficulties faced by people with MS. COB-MS is a step-by-step evidence-based intervention that can be facilitated by occupational therapists and enable people to identify, understand, and implement strategies to simultaneously manage cognitive impairment and improve function in daily life.

To conclude, this pilot study established the clinical outcomes of COB-MS in relation to daily life and cognition. This study provides foundational evidence to support COB-MS as the most suitable intervention for people with MS who experience cognitive difficulties in daily life and adds to the paucity of research in the field of MS and CR.

## Figures and Tables

**Table 1:** 

Session	Brief content
Session 1 (individual)	*Focus on you* (i) Initial meeting with the OT: briefing on what will be involved in COB-MS (ii) Goal setting with the person on occupations that they wish to target

Session 2 (group)	*You and your cognition* (i) Education about the brain and cognition (ii) Discussion on how MS can impact cognitive function (iii) Discussion on the impact of cognitive difficulties on day-to-day occupations

Session 3 (group)	*You, the centerpiece* (i) How the cognitive difficulties affect you? (ii) What changes can be made by you? (iii) Discussion and application of strategies

Session 4 (group)	*You, the person* (i) Further concepts on what changes can be made by you (ii) Internal strategies to practice and apply (iii) Discussion around sleep and sleep diaries

Session 5 (group)	*Your environment* (i) How does the environment impact cognition? (ii) What can we change that might help? (iii) Application of external memory strategies (iv) Managing distraction and the impact of other factors

Session 6 (group)	*Focus on doing* (i) How are our occupations and daily life affected? (ii) What can we do to help integrate strategies into daily life? (iii) Examples of how to adapt or remediate occupations

Session 7 (group)	*Seeking new challenges* (i) Seek new challenges with strategies that support success (ii) Set goals for yourself (iii) Stay motivated, maintain progress and on-going adaptation (iv) Group conclusion and debrief

Session 8 (individual)	*Testing the application* (i) Review goals and strategies used (ii) Plan for future (iii) Discussion around useful groups and services (iv) Debrief and summary

**Table 2:** Demographic data of the sample.

	Mean	SD	Minimum	Maximum
Age (years)	55.08	9.61	39	73
Duration (years)	14.25	7.61	3	31
Education (years)	16.42	2.31	13	20
MSNQ	26.58	2.27	23	30

**Table 3:** Wilcoxon signed-rank test results for the GAS.

	Time 1			Time 3			*z*-value	*p* value
	Mean	SD	Median	Mean	SD	Median		
GAS	37.02	0.88	37.5	56.23	10.60	52.7	−3.061	.002^*∗*^

^*∗*^ *p* value is statistically significant at *p* < .05.

**Table 4:** Friedman test results for the OSA-DLS.

	Time 1			Time 2			Time 3			*p* value
	Mean	SD	Median	Mean	SD	Median	Mean	SD	Median	
Competence	30.58	7.50	31.00	31.67	6.77	33.00	31.33	7.55	34.00	.822
Importance	31.42	6.54	31.00	31.92	8.59	35.00	31.67	8.19	31.00	1.000

**Table 5:** Friedman test results for cognitive outcome measures.

	Time 1			Time 2			Time 3			*p* value
	Mean	SD	Median	Mean	SD	Median	Mean	SD	Median	
*CVLT-II*										
Total free recall	45.92	6.83	45.00	58.00	7.10	56.00	54.50	9.85	52.00	.000^*∗*^
Short delay free recall	9.00	2.09	9.00	12.17	2.41	13.00	11.25	3.22	13.00	.018^*∗*^
Long delay free recall	9.67	2.81	10.00	12.33	2.06	13.00	11.58	2.84	12.00	.008^*∗*^
*BVMT-R*										
Total recall	45.67	9.87	44.00	51.33	12.61	52.00	57.33	10.22	60.00	.000^*∗*^
Delayed recall	48.83	12.77	49.00	52.42	14.87	56.00	57.58	8.22	61.00	.103
*SDMT*	38.42	8.08	38.00	39.83	10.20	37.00	41.75	9.28	37.00	.127
*TMT*										
Part A	35.92	12.07	31.00	34.33	11.51	33.00	33.75	15.39	33.00	.368
Part B	95.92	47.96	79.00	69.17	31.57	56.00	67.33	27.58	62.00	.044^*∗*^
*BRIEF-A*										
Behavioural regulation index	58.83	9.71	58.00	57.58	8.55	60.00	59.33	7.98	61.00	.376
Metacognition index	61.58	11.31	61.00	61.08	7.83	63.00	63.00	7.94	63.00	.862
Global executive composite	61.17	10.05	63.00	60.25	8.01	62.00	61.25	8.21	61.00	.320
*EMQ-R*	19.83	10.85	18.00	15.67	12.46	14.00	16.00	11.07	14.00	.006^*∗*^

^*∗*^ *p* value is statistically significant at *p* < .05.

**Table 6:** Wilcoxon signed-rank test results for post hoc tests.

	Time 1	Time 2	Time 3
*CVLT-II*: total free recall	.002^*∗*^	.181	.003^*∗*^
*CVLT-II*: short delay free recall	.008^*∗*^	.058	.052
*CVLT-II*: long delay free recall	.012^*∗*^	.311	.044
*BVMT-R*: total recall	.028	.012^*∗*^	.022
*TMT*: part B	.077	.610	.016^*∗*^
*EMQ-R*	.040	.563	.022

^*∗*^ *p* value is statistically significant at *p* < .0167.
